# The Impact of Disclosure on Health and Related Outcomes in Human Immunodeficiency Virus-Infected Children: A Literature Review

**DOI:** 10.3389/fpubh.2017.00231

**Published:** 2017-08-30

**Authors:** Angela Odiachi

**Affiliations:** ^1^Department of Health Policy and Management, University of North Carolina at Chapel Hill, Chapel Hill, NC, United States

**Keywords:** human immunodeficiency virus, children living with human immunodeficiency virus, pediatric disclosure, health outcomes, review

## Abstract

This review explores the association between pediatric human immunodeficiency virus (HIV) disclosure and health and related outcomes among children living with HIV. A multi-stage process was used to search for relevant articles on the ISI Web of Knowledge database. Fifteen articles met the inclusion criteria. Five major outcomes emerged from children’s knowledge of their HIV-seropositive status: physical/physiological outcomes; adherence to antiretroviral therapy; psychosocial outcomes; sexual and reproductive health, including HIV prevention outcomes; and disclosure of status by the children. Disclosure of a child’s HIV status to the child has value in terms of positive health outcomes for the child, such as better adherence and slower disease progression—albeit the different studies did not always reach the same conclusions, and some suggest negative health outcomes, such as increased psychiatric hospitalization. Yet, there does not seem to be a systematic or coherent system for child disclosure. One recommendation from this review, therefore, is for government and program policies and guidelines that will promote child HIV disclosure in order to address the current low rates of disclosure in sub-Saharan Africa (SSA). More rigorous and longitudinal studies on the outcomes of disclosure, with larger sample sizes, and in SSA, are also needed.

## Introduction

More than 80% of the estimated 1.8 million children <15 years infected with human immunodeficiency virus (HIV) in 2015 lived in sub-Saharan Africa (SSA) (1). The global initiative—*The Global Plan toward the elimination of new HIV infections among children by 2015 and keeping their mothers alive (Global Plan)*—was launched in 2011 at the United Nations General Assembly High Level Meeting on acquired immune deficiency syndrome (AIDS) (2). The plan prioritized the 22 countries that in 2009 accounted for 90% of the global mother-to-child HIV transmission burden: 21 of the Global Plan priority countries were in SSA. Among other things, the plan sought to increase the coverage of prevention of mother-to-child transmission of HIV (PMTCT) interventions and reduce the incidence of new infections in children. At the end of the initiative in 2015, there were remarkable results as a result of significant increases in access to antiretroviral (ARV) drugs, with six priority countries meeting the Global Plan goal of 90% ARV coverage for pregnant women living with HIV; a reduction in final mother-to-child transmission rates to 8.9% from 22.4% in 2009 (with four countries reaching the milestone of <5% transmission); and 60% decline in new infections in children in the 21 SSA priority countries (3). Despite this progress, PMTCT programs in resource-limited countries are still fraught with challenges. Consequently, many children continue to be infected perinatally with HIV. In 2015, there were 150,000 new HIV infections in children 0–14 years globally. Of these, 122,000 (> 80%) were in SSA (1). Data suggest that 60% of these new infections may be happening during breastfeeding, due to poor treatment adherence and systems for following up breastfeeding HIV-positive women and their babies (2). However, as a result of increased ARV availability to these children, they are living longer (4). Thus, a generation of children living with HIV (CLHIV) is coming of age. As these children approach adolescence, many of them have not been disclosed to. The term *disclosure*, in this context, refers to informing children that they have HIV.

The World Health Organization *Guideline on HIV Counseling for Children up to 12 years of Age* recommends that children of school age (6–12 years) be told they have HIV (5). The American Academy of Pediatrics also recommends HIV status disclosure to school aged children (6). Disclosure prevalence from four studies in developing countries ranged from 29 to 62% (4). Vaz et al. (4) reported only 3% pediatric disclosure in their study in the Democratic Republic of Congo, while Vreeman et al. (7) also reported almost 100% non-disclosure in Kenya. More recent studies in SSA have similarly reported low disclosure rates—13.5% (8) and 30.9% (9) in Nigeria; 21% (10) in Ghana; 17.4% (11) and 39.5% (12) in Ethiopia; 19% (13) and 26% (14) in Kenya; and 32.6% (15) in Cote d’Ivoire.

Factors that influence pediatric disclosure include child’s age and cognitive development (10, 12); concerns around antiretroviral therapy (ART) adherence (8, 9); imminent onset of sexual activity (4); and the need to protect others from infection (16). Benefits of pediatric disclosure include improved adherence to ART, and psychosocial well-being and mental health (17). Despite these benefits of disclosure, non-disclosure remains high because of the association of HIV-positive status and promiscuity in parents (18). Disclosure of HIV status to CLHIV or to adult partners remains “navigation in a moral field” (18). Therefore, to protect the family name and one’s reputation, and avoid rejection and discrimination, many parents choose not to disclose HIV status to children (18). Other reasons for non-disclosure include caregivers’ concerns that children were too young (9, 10) and caregivers’ fear of the psychological impact of disclosure on the children (8, 12).

Literature on disclosure suggests that when disclosure does happen, it is not done in a systematic way (4, 19). The process remains largely context dependent. Also, many SSA countries do not have clear and detailed policies and guidelines on pediatric disclosure. Yet, disclosure could be a potent force in the prevention and control of HIV infection to those not infected (4, 20). And for those who are already infected, it provides an opportunity for improved quality of life (QoL) for the HIV infected and their families, slowing of disease progression (4).

This literature review, therefore, explores the association between pediatric disclosure, i.e., disclosure of child’s seropositive HIV status, and health outcomes among CLHIV: is there any correlation between HIV disclosure and improved or worsened health—physical, psychological, or other dimensions of health? While the review looked at pediatric HIV disclosure in all contexts, particular interest was on SSA, since most CLHIV reside in this part of the world (21). This review focuses solely on the impact of disclosure, unlike other reviews (22, 23), which focused on themes such as process, prevalence, impact, and other aspects of disclosure. As such, this review looks at disclosure outcomes in more detail and highlights these findings in-depth, as a result of its single focus.

## Methods

### Search Process

A multi-stage process was used to search for data on disclosure of HIV status to CLHIV in 2011. Articles pertinent to the research question, “The Impact of Disclosure on Health Outcomes for HIV-Infected Children,” were searched for in the ISI Web of Knowledge database, using the terms arrangement as follows: ((Child* OR adolescent OR p*diatric OR perinatal*) AND (HIV OR status) AND (Diclos*)). This database was expected to provide a robust number of search findings. The search strategy was repeated in 2014 for additional peer-reviewed articles that may have been published since the last search. This second search was limited to studies conducted in SSA, since that was the region of immediate interest. This decision was informed by the assumption that more readily applicable research findings would likely come from similar SSA settings.

### Inclusion Criteria

Only articles on studies published in peer-reviewed journals were included in the review. Articles had to focus on disclosure of HIV status to children (persons under 18 years) living with HIV and be based on primary data collection. Since it was anticipated that there would be a wealth of available primary data on the subject (and there were) systematic reviews or meta-analyses were not included in the review. Commentaries were also not included in the review. Studies could be qualitative or quantitative, or both. However, they had to contain an explicit definition of the term disclosure or a clear indication that children knew their positive HIV-serostatus, and the consequences and outcomes of such disclosure as a dependent or independent variable. Studies could focus on only children to whom their status had been disclosed to them, or also contain a control group whose status was not disclosed to them. The most important element was that studies were limited to those where full disclosure of HIV status was done. “A child was considered to be fully informed of his or her status if the term HIV, AIDS, or any local term specifically associated with HIV/AIDS has been used in a discussion with the child about the child’s health”—[page 248 of Ref. (4)]. Reviewed articles also had to include a clear description of the population size, data collection process, the independent and dependent variables (for quantitative studies), how data were analyzed, and the main themes from data analysis (for qualitative studies).

### Exclusion Criteria

Disclosure studies of HIV status of others—adults and parents—were not included. Only studies where disclosure was by a parent, caregiver, or health care provider were included. Studies where children learned of their serostatus inadvertently through other sources were not included in the review, as it is believed that the effect of such disclosure may be different from that through a controlled environment through a parent/caregiver or health provider.

Studies where there was only partial disclosure, i.e., discussing with children about the child’s health in general terms, without specific mention of HIV or AIDS, and non-English language articles were not included. There was no time limit or country or regional restriction to the studies or publications included in the review from the first search. However, the second search was limited to only SSA studies.

### Identified Studies

The initial search in 2011 yielded a total of 426 articles. After a review of the article titles, 242 articles that were not relevant to the research question were eliminated from further search. Abstracts for the remaining 184 articles were reviewed, after which a further 144 articles were excluded because of content (135), three were in French, and the rest were editorials, articles, and letters. Another three articles could not be retrieved from the UNC library. No further attempts were made to retrieve the articles.

**Figure 1:**
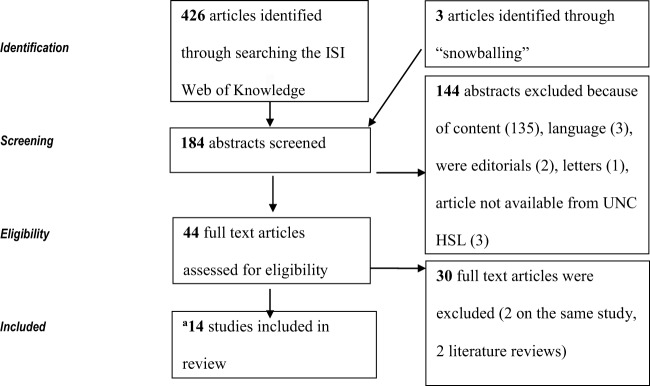
Flowchart of the identification of studies included in the review.

Full text of the 44 articles that appeared relevant to the research question was then reviewed for eligibility. Fifteen articles from this initial search in 2011 met the inclusion criteria, but two articles were publications on the same study, so one was eliminated from further review (Figure 1).

An additional eight studies were identified from the 2014 search. While all reported disclosure rates and factors that affected status disclosure, only one study assessed the association between disclosure and health outcomes, namely ART adherence, and stigma and depression (14), and was included in the review, making a total of 15 articles that were included in the review (Table 1).

**Table 1:** Effect of human immunodeficiency virus (HIV) disclosure on health and related outcomes.

#	Effect of disclosure on health outcomes	Reference	Study goal	Country	Sample size	Study type	Study design	Type of analysis	Independent variable	Dependent variable	Results
1	Mental and psychosocial outcomes; Sexual and reproductive health outcomes; Child’s disclosure of status to others	Battles and Weiner (29)	Examine psychosocial factors associated with long-term survival of pediatric HIV infection	USA	80 parent–children living with HIV (CLHIV) dyads	Quantitative	Descriptive longitudinal study	Pearson product moment relations, Chi-square, Student’s *t* tests	HIV disclosure Child’s disclosure of status to others	Social support, child (problem) behavior, self-perception, competence	Of 67 disclosed CLHIV, 4 had psychiatric hospitalization, 19 clinical anxiety, and 25 clinical depression 21 disclosed CLHIV were sexually active—with or without condom use Pearson product moment correlations showed that disclosure was positively related to social support (*r* = 0.35, *p* < 0.05), self-competence (*r* = 0.35, *p* = 0.08), decreased problem behavior (*r* = −0.21, *p* = 0.08). However, public disclosure (i.e., disclosure through television and newspaper), showed negative association with global self-competence (*F* = 3.5, *p* < 0.05)
2	Sexual and reproductive health outcomes; Child’s disclosure of status to others	Birungi et al. (20)	Examine sexual expressions and experiences and preventive practices and needs of CLHIV	Uganda	732 CLHIV 4 health care workers (HCWs)	Qualitative and quantitative	Cross-sectional study	Quantitative: cross tabulations, Chi-square, significance tests of proportions; qualitative: content analysis	HIV disclosure	Condom use, contraceptive use; HIV status disclosure to others	47% of (disclosed) CLHIV who had ever had sex reported currently using condoms, compared to general adolescent population (15–19 years) who had ever had sex, where 44% had ever used any form of contraception and only 11% reported currently using condoms 49% of 158 CLHIV in a current relationship had disclosed to their partners
3	Adherence to treatment; Mental and psychosocial outcomes; Child’s disclosure of status to others	Blasini et al. (27)	Describe effect of disclosure on health care professionals, caregivers, and HIV-infected youth	Puerto Rico	40 CLHIV 39 caregivers 16 HCWs	Quantitative	Quasi experimental design (comparison of before and after disclosure).	Fisher’s exact test	HIV disclosure	Sadness, worry, insecurity, and other psychosocial outcomes	42% CLHIV felt sad immediately after disclosure. At 6 months, 70% youth reported normalcy. One patient reported depression after 6 months 62% CLHIV chose not to disclose their status to others. 38% CLHIV disclosed to close family (siblings and cousins) 58% CLHIV reported better adherence to treatment after disclosure
4	Physical/physiological outcomes; Mental and psychosocial outcomes	Butler et al. (26)	Examine impact of disclosure on health-related pediatric quality of life (QoL) and describe distribution of age at disclosure	USA	395 CLHIV (2,423 study visits)	Quantitative	Prospective cohort study (PACTG 219 C, comparison of QoL domains before and after disclosure)	Wilcoxon signed rank tests, multivariate mixed-effects model	HIV disclosure	Six QoL domains: (general health perception, symptom distress; psychological status, physical functioning; social/role functioning, and health care utilization)	In mixed-effects models, disclosure did not significantly impact QoL for any domain when comparing before and after disclosure: General health perception (*p* = 0.70); symptom distress (*p* = 0.31), psychological status (*p* > 0.999), physical functioning (*p* = 0.79); social/role functioning (*p* = 0.69); health care use (*p* = 0.61)
5	Mental and psychosocial outcomes; Child’s disclosure of status to others	Campbell et al. (33)	Investigate impact of HIV transition program on participants’ lives	UK	6 CLHIV	Qualitative	Individual interviews (by telephone or in-person)	Thematic approach	HIV disclosure	Disclosure to others; hopes for the future	CLHIV were hopeful about the future However, they expressed concerns about disclosure to romantic/sexual partners
6	Physical/physiological outcomes	Cohen et al. (25)	Describe issues related to school attendance and HIV disclosure to CLHIV	USA	92 CLHIV	Quantitative	HIV surveillance, cross-sectional clinic survey, abstraction of medical records	χ*^2^*, logistic regression	HIV disclosure	Clinical severity of symptoms (CDC categories—mild, moderate, and severe)	Clinical severity of child’s symptoms was not associated with child’s knowledge of status. 49% of children with severe symptoms were disclose, compared with 39% of children with mild and moderate symptoms
7	Physical/physiological outcomes	Ferris et al. (24)	Investigate disclosure effect on disease progression (death, CD4 decline)	Romania	325 CLHIV	Quantitative	Retrospective database analysis (comparison of disclosed vs. non-disclosed children)	Student t tests, Chi-square test, Fischer’s exact test, Cox regression models	HIV disclosure	Death, CD4 decline, combined, time to HIV disease progression	Non-disclosed children were more likely to experience disease progression through either death or CD4 decline than children who knew their HIV diagnosis (*p* = 0.03)
8	Mental and psychosocial outcomes	Gaughan et al. (28)	Determine the incidence of psychiatric hospitalizations among CLHIV and predictors of first psychiatric hospitalization	USA	2,298 CLHIV, 1,021 non-CLHIV	Quantitative	Prospective cohort study (PACTG 219 C)	Relative risks using Poisson rate parameters; Cox proportional hazards regression	HIV disclosure	Psychiatric hospitalization	CLHIV who were aware of their status were six times more likely to be hospitalized due to psychiatric illnesses, compared to CLHIV not aware of their status (hazard ratio 6.13)
9	Mental and psychosocial outcomes	Lester et al. (30)	Determine factors related to timing and probability of non-disclosure of HIV status to CLHIV and factors associated with emotional distress in CLHIV	USA	51 CLHIV, 49 parents	Quantitative	Cross-sectional study	Content data analysis; Kaplan–Meier, Cox proportional hazards model, multiple linear regression	HIV disclosure	Anxiety and depression in children—rated separately by children and their parents	Parents’ ratings of children’s anxiety show an association between HIV disclosure and greater child anxiety (*t* = 2.15, *p* = 0.04). However, children’s own reports of anxiety and depression did not show corresponding elevations in distress in relationship to HIV disclosure
10	Mental and psychosocial outcomes	Menon et al. (32)	Examine emotional and behavioral difficulties in HIV-positive adolescents, and relationship between HIV disclosure and mental health	Zambia	127 CLHIV–caregiver dyads	Quantitative and qualitative	Cross-sectional survey	Mann–Whitney *U* test, χ*^2^*, Spearman, content analysis	HIV disclosure	Strengths and difficulties questionnaire (SDQ), scores for emotional symptoms, conduct problems, hyperactivity/inattention, peer relationship problems, and prosocial behavior	Univariate analyses showed no differences in continuous SDQ-Y scores between disclosed and non-disclosed children. However, fewer disclosed CLHIV had extreme scores for emotional difficulties (18.8 vs. 38.8%, χ*^2^* = 4.1, df = 1; *p* = 0.04) Non-disclosed CLHIV were twice as likely to experience concerning levels of emotional difficulties as disclosed CLHIV (OR = 2.63, 95% CI: 1.11–6.26)
11	Adherence to treatment; Mental and psychosocial outcomes; Child’s disclosure of status to others	Petersen et al. (17)	Understand psychosocial challenges and protective influences that promote socio-emotional coping in HIV+ adolescents	South Africa	25 CLHIV, 15 caregivers	Qualitative	Individual interviews (in-person)	Thematic analyses	HIV disclosure	Identity, psychosocial issues, internalized stigma	All 25 adolescents reported good adherence 22 CLHIV reported that knowing their status was emotionally difficult; 9 CLHIV (36%) withdrew from friends, as a result of difficulty in accepting an HIV+ identity. 13 CLHIV (>50%) showed internalized stigma CLHIV expressed concerns about how to negotiate future sexual relationships. Also, only 13 CLHIV had disclosed their status beyond the immediate family
12	Child’s disclosure of status to others	Sherman et al. (34)	Examine physiological and psychological consequences of children’s self-disclosure	USA	64 CLHIV–caregiver dyads	Quantitative	Comparison of CLHIV who had self-disclosed their status (disclosers) to CLHIV who had not (non-disclosers)	Univariate ANOVA, χ*^2^*, Tukey’s honestly significant difference tests	Child’s self-disclosure	Child’s CD4% (disease progression); self-concept, behavioral problems (psychological well-being)	CLHIV who disclosed their HIV status to friends had a significantly larger increase in CD4% (mean = +5.55, SD = 5.92), implying a slowing of disease progression, relative to non-disclosers (mean = 0.00, SD = 5.75); ANOVA *F*(2,60) = 4.28, *p* < 0.05 ANOVA analysis of changes in self-concept did not approach significance between disclosers and non-disclosers, *F*(2,60) = 0.56, *p* > 0.15 ANOVA analysis of changes in levels of behavioral problems (comparing disclosers and non-disclosers) also did not approach significance, *F*(2,57) = 0.69, *p* > 0.15
13	Mental and psychosocial outcomes	Sopena et al. (31)	Identify if CLHIV had poor psychological adjustment and clarify relation- ship between coping and psychological adjustment in CLHIV	UK	30 CLHIV	Quantitative	Correlational design (comparison of disclosed CLHIV and general British population)	*t*-test, Pearson correlations	HIV disclosure	Total strengths and difficulties, SDQ, score on psychological subscales (emotional, conduct, inattention-hyperactivity, peer problems, and prosocial); coping behaviors	Disclosed CLHIIV did not exhibit problems with psychological adjustment as measured by SDQ scores No significant difference between disclosed CLHIV and general UK population: Psychological adjustment total SDQ score *t*(29) = −1.03. *p* > 0.05
14	Mental and psychosocial outcomes	Vaz et al. (4)	Explore events before, during and after disclosure	Democratic Republic of Congo	8 CLHIV–caregiver dyads	Qualitative	Individual interviews (in-person)	Content analysis	HIV disclosure	Disclosure experiences and reactions	Children felt sad immediately after disclosure. But later did not state any negative effect of knowing their status. Benefits of disclosure included relief, not being worried and avoiding being sicker; and being able to protect others
15	Physical/physiological outcomes; Adherence to treatment; Mental and psychosocial outcomes	Vreeman et al. (14)	Assess association between disclosure and key child level demographic, clinical, and psychosocial characteristics	Kenya	792 caregiver–CLHIV dyads	Cross-sectional, quantitative	Comparison of disclosed vs. non-disclosed children, medical chart review	Pearson’s Chi-square test, multivariate logistic regression with odds ratio	HIV disclosure	Clinical characteristics—adherence, CD4 count, CD4%, WHO staging; psychosocial characteristics (stigma, depression)	No association between disclosure and WHO staging (*p* = 0.079), and CD4% (*p* = 0.582) Disclosure was associated with child-reported adherence (*p* = 0.03) Caregiver-reported child-experienced stigma and child depression symptoms were both significantly associated with disclosure (*p* < 0.01)

Due to the limited number of studies that met the inclusion criteria, the inclusion of articles did not focus on their internal validity based on the study approaches, strong statistical power, or an experimental approach. Nor was the external validity of articles a limiting factor in terms of a large study population, random sample, and explicit analysis of context and intervention factors for which generalization is possible. (The impact is discussed under the Discussion section, as a limitation of the studies in this review.)

### Data Extraction

Information on authors, year of article, and country where study was conducted, participant characteristics (study participants, children’s age), and study characteristics (sample size, study type and design, type of analysis, dependent and independent variables, results, statistics, significance, and study validity information), and the health outcomes of disclosure were extracted from the studies (see Table 1).

## Results

Five major health and related outcomes emerged from children’s knowledge of their seropositive status (Table 1): disease progression (CD4 count, death) and other physical/physiological outcomes; adherence to ART; self-esteem, mental, emotional, and other psychosocial outcomes; and sexual and reproductive health (SRH), including HIV prevention outcomes. The latter was particularly relevant to another theme that emerged from the results that was not in the original review conceptualization—disclosure of status by the children to friends and sexual partners.

### Physical/Physiological Outcomes

Four studies described the physical/physiological health outcomes of status disclosure in CLHIV. The first, a comparison study of 325 Romanian children aged 5–17 years on ART, some of whom had been told their serostatus and others who were non-disclosed, showed a significant difference in disease progression as measured by decline in CD4 count and death (24). A Kaplan–Meier survival analysis showed that non-disclosed children were more likely to die (*p* = 0.03). Although there was no significant difference in CD4 decline, a greater proportion of non-disclosed children experienced CD4 decline (*p* = 0.26) and were more likely to experience death than children who knew their status (*p* = 0.03).

A 1997 multicentre Pediatric Spectrum of Disease active surveillance study of 92 American school CLHIV in Massachusetts, however, did not show any association between clinical severity of children’s symptoms (CDC clinical stage of mild, moderate or severe) with whether a child was told of his or her disease status (25). Forty-eight percent of children with severe symptoms had been told of their status compared to 39% of children with mild to moderate symptoms. Cohen’s 1997 study seems to suggest that if a child had severe symptoms they were more likely to know their status, even if this was not statistically significant.

Butler et al. (26) reported that there were no significant changes in physical functioning, or health care utilization domains between pre-disclosure and post-disclosure in their PACTG QoL study of 395 perinatally HIV-infected youth. Similarly, Vreeman et al. (14) did not find any associations (in multivariate analysis) between disclosure status and clinical indicators, like CD4% (*p* = 0.582) and WHO disease stage (*p* = 0.079) in their study of 792 caregiver–child dyads in Kenya.

### Adherence to Treatment

Three studies focused on the effect of disclosure of child’s status to the child and treatment adherence. The quasi experimental study of disclosure’s effect on 40 children on ART in Puerto Rico showed that over half (58%, 95% CI 41–73%) self-reported that knowing their status had helped them develop better adherence to their medicines (27). All 25 adolescents and their caregivers in the South African qualitative study reported good adherence as a result of the children knowing their status (17). These adolescents reported adherence to treatment as a positive coping strategy for their HIV+ status, as such adherence would help them live longer. In the Kenya study by Vreeman et al. (14) disclosure status was not associated with adherence as reported on the clinical encounter form or by caregivers. However, disclosure was associated with child-reported adherence (*p* = 0.03) and disclosed children reported more non-adherence than non-disclosed children.

### Mental and Psychosocial Outcomes

Majority of the studies reviewed focused on the mental, emotional and other psychosocial effects of disclosure, since this is one of the reasons often cited for both disclosure and non-disclosure to children. Eleven articles, four of which were in SSA, focused on this health outcome (see Table 1). While two of the articles were on the same Pediatric AIDS Clinical Trials Group (PACTG) 219 C prospective cohort study (26, 28), the authors and foci of the two articles were different and were therefore included as separate studies in this review. The first PACTG 219 C study focused on the effect of HIV disclosure on the QoL based on 2,423 study visits by 395 CLHIV in USA (26). The study showed that there were no statistically significant differences between pre-disclosure and post-disclosure QoL domains (general health perception, symptom distress, psychological status, health care utilization, physical functioning, and social/role functioning). Disclosure was not significantly associated with QoL in crude or adjusted mixed-effects model analyses, indicating that QoL did not change because of disclosure of HIV infection status. Caregivers reported lower QoL scores after disclosure for all domains except social/role functioning, although these differences were not significant. The other PACTG 219 C study, however, reported that CLHIV were at increased risk of psychiatric hospitalization than the general pediatric population, and knowledge of seropositive status was significantly associated with increased risks of admission in this population (28). Multivariate analysis showed that CLHIV who were aware of their status were six times more likely to be hospitalized because of psychiatric illnesses compared to those who were not, mostly for depression and behavioral disorders—which are precursors for more severe pathologic conditions, such as bipolar disorder and suicide. Battles and Wiener (29) also reported that four of 67 disclosed CLHIV (≥13 years) in their US study had been hospitalized for psychiatric illness. In addition, 19 and 25 CLHIV had received a clinical diagnosis for anxiety and depression, respectively, by a psychiatrist. Four CLHIV had also attempted suicide. The authors, however, did not indicate how these numbers compared with the general US adolescent population, or non-disclosed CLHIV.

The progression of patients’ self-reported emotions after disclosure ranged from sadness immediately after disclosure to normalcy by most youth (70%, *N* = 40, *p* < 0.05) after 6 months of disclosure (27). However, one patient remained depressed 6 months after disclosure and wished he had never learnt of his status (27). A majority (85%) of the children they felt disclosure was a positive event for them and their family. While 90% of the children favored disclosure, 10% did not. Incidentally the 10% who did not favor disclosure, learnt of their status accidentally, and wished that they had learnt of their serostatus from family or health care workers. Lester et al. (30), however, suggest in their US study of 51 CLHIV that disclosure of status may not necessarily minimize emotional distress in children, as HIV disclosure was associated with increased anxiety in HIV-infected children reported by parents (*p* = 0.04). Interestingly, the children’s own report did not show corresponding increases in anxiety and depression in relation to HIV disclosure.

A UK study of 30 disclosed CLHIV did not show any statistical difference in psychological (emotional and behavioral) adjustment than the general population, as measured by the strengths and difficulties questionnaire [total SDQ score *t*(29) = −1.03. *p* > 0.05; SDQ score of 0.56 which approaches acceptability levels] (31). However, a similar study in Zambia using the same SDQ methodology showed increased mental health problems (OR = 2.1), especially emotional symptoms (OR = 3.6) and peer problems (OR = 7.1) than the UK sample (32). Univariate analysis showed no difference between children who knew their HIV status and those who were non-disclosed. However, there were fewer participants in the disclosed group with extreme scores in the borderline or abnormal range for emotional difficulties (18.8 vs. 38.8%, χ*^2^* = 4.1, df = 1; *p* = 0.04); and non-disclosed children were twice as likely to experience emotional difficulties (OR = 2.63, 95% CI: 1.11–6.26) than disclosed children (32).

The South African study by Petersen et al. (17) showed similar emotional difficulties for children who received disclosure of their positive HIV diagnosis. Thirty-six percent (*N* = 9) reported withdrawing from their friends and social activities, as a result of the difficulty they experienced in accepting an HIV+ identity. Over 50% reported internalized stigma. But for the eight children in an exploratory study in the Democratic Republic of Congo who knew their status, despite the negative emotions experienced at the time of disclosure (such as sadness and worry), there were no subsequent negative effects of knowing their status (4). For them, the benefits of knowing their status included relief, no longer worrying (so they could avoid being sicker), and being able to protect others from HIV infection.

The Kenya study of 792 caregiver–CLHIV dyads found that in univariate analysis, there was a significant association between disclosure and caregiver-reported child-experienced stigma (*p* < 0.01) and child depression symptoms (*p* < 0.01) (14). While 2% caregivers of non-disclosed children reported stigma and 4% reported depression symptoms, 10% of caregivers of disclosed children reported stigma and 12% reported depression symptoms in their children. However, only depression symptoms were significantly associated with disclosure in multivariate regression (OR = 2.6, 95% CI 1.1–6.2) (14).

A small-scale qualitative study of six program participants at a transition to adulthood program in the UK showed that participation in the transition program facilitated a positive attitude toward medication and hope for the future in disclosed CLHIV (33).

### Sexual and Reproductive Health

Young CLHIV receive health services under pediatric care and are often not being adequately prepared for adult life (20). Two studies focused on SRH issues for CLHIV (20, 29). In terms of SRH services, especially in relation to preventive practices, such as condom or contraceptive use among sexually active CLHIV, only 37% (*N* = 236) of CLHIV in a Population Council study in Uganda reported using a condom at time of first sex (20). Only 50% used any form of contraception in current or previous relationships, and 47% reported current condom use. (All figures were statistically significant, *p* < 0.05.) These are relatively high use rates compared to the general population, and especially for adolescent population (20). While this may seem to suggest more careful behavior by CLHIV, other findings from the study paint a different picture: only a third of CLHIV currently in a relationship knew the HIV status of their partner. Also, there was no significant difference in the use of condoms by CLHIV who knew the status of their sexual partner and those who did not (57 vs. 58%).

Battles and Wiener (29) reported in their US study that two (5.3%) of 40 disclosed CLHIV (13–17 years) and 19 (70.4%) of 27 disclosed CLHIV (≥18 years) were sexually active—with or without using condoms. There was no information on how these children compared to the general US population or non-disclosed children.

### Disclosure of Status to Others by Children

Parents of CLHIV worry about whether to let their children disclose their (CLHIV) status to others, usually because of fear of stigma and discrimination (9, 34). However, research has shown that self-disclosure of traumatic or secretive information produces observable health benefits (34). In this regard, the focus is on the extent of self-disclosure, and whether such self-disclosure influences health outcomes, such as the immune response, psychological well-being, and other health outcomes. Six studies on this issue met the inclusion criteria (17, 20, 27, 29, 33, 34). In the US study on 64 CLHIV–caregiver dyads, Sherman et al. (34) showed that CLHIV who knew their positive HIV status and had in turn self-disclosed their HIV status to their friends over the past year (recent disclosers), had a significantly higher CD4% (mean = +5.55, SD = 5.92) than CLHIV who had not self-disclosed (mean = 0.00, SD = 5.75) (ANOVA *F*(2,60) = 4.28, *p* < 0.05), implying a slower disease progression in disclosed CLHIV. Psychological well-being, as measured by self-concept for disclosers vs. non-disclosers, did not approach significance [*F*(2,60) = 0.56, *p* > 0.15]. Similar ANOVA analysis for changes in behavioral problems also did not approach significance [*F*(2, 5) = 0.69, *p* > 0.15].

Battles and Wiener (29) conducted semi-structured interviews with disclosed CLHIV as part of their US long-term pediatric HIV survival study on 80 parent–child dyads, in order to assess the degree of disclosure to others of the child’s diagnosis and whether such disclosure had an effect on psychosocial outcomes. Pearson product moment correlations showed that disclosure was positively related to social support (*r* = 0.35, *p* < 0.05), self-competence (*r* = 0.35, *p* = 0.08), and decreased problem behavior (*r* = −0.21, *p* ≤ 0.08). However, for public disclosure (i.e., disclosure to the media—television and newspapers), Student’s *t*-test showed a negative association with self-competence (*F* = 3.5, *p* < 0.05). In other words, greater disclosure was associated with increased social support, social self-competence, and decreased problem behavior, but public disclosure was associated with lower self-competence (29).

In the Puerto Rico study by Blasini et al. (27), 62% of the 40 children in the study chose not to disclose their status to others. The remaining 38% who disclosed, did so to family members—siblings and cousins. Only three of them disclosed to a close friend.

Another aspect of disclosure of status to others relates to disclosure to sexual partners. That is, how knowledge of one’s status prompts disclosure to sexual partners, or not, as disclosure could prompt the adoption of HIV prevention strategies in the relationship. A Uganda study (20) showed that only 77 of the 158 adolescents (i.e., 49%) in a relationship had disclosed to their partners. Respondents in a small qualitative study based on a UK transition program reported not disclosing their status to others, including sexual partners (33). They expressed their concerns about status disclosure in their romantic/sexual relationships, and therefore, the importance of meeting other HIV+ young people. The adolescents in the South Africa study by Petersen et al. (17) also expressed concerns about how to negotiate future heterosexual relationships, wondering how they would disclose to future partners. Furthermore, only 13 of the 25 adolescents in the study had disclosed to persons beyond their immediate family for fear of stigma and discrimination. Such disclosure was usually to a school teacher (so as to receive academic support), or a friend.

## Discussion

### Findings

One major health outcome of HIV disclosure was ART adherence. Since ART is life long, one recurring challenge for caregivers and CLHIV is how to maintain treatment adherence. With ART, a high adherence level of up to 95% or more is necessary to avoid drug resistance and its very serious consequence of treatment failure (35). As such ART adherence is a critical factor in managing HIV infection. One would, therefore, have expected more studies on the effect of disclosure on CLHIV treatment adherence, since this is the reason most often given for promoting status disclosure. However, only three studies focused on the effect of disclosure of child’s status to the child and treatment adherence (14, 17, 27). As expected, the children and their caregivers reported improved adherence to treatment as a result of the children knowing their HIV status. Incidentally, the small sample sizes (40 and 25) (17, 27) and the less than rigorous analysis limit any broad conclusions on the impact of disclosure on treatment adherence. This review, therefore, calls for more studies considering the importance of adherence on HIV treatment for CLHIV.

A second major finding from this review is that HIV disclosure to CLHIV appears to be associated with disease progression in terms of clinical severity of symptoms, CD4 percent and ultimately death. While the Romanian comparison study (24) showed that HIV disclosure was associated with a slowing down of disease progression through higher CD4 cells, the US study did not show any impact of HIV disclosure on clinical severity of disease symptoms (25), or did the Kenya study show any association with CD4 count (14). It may be argued that the US study used a limited sample size, and no information on the statistical significance of the results was presented compared to the more rigorous analysis of the Romanian study, which included adjusting for confounders. All the same, more prospective studies on larger sample CLHIV populations are needed to draw any definitive conclusions on the effects of HIV disclosure on disease progression and severity.

Understandably, majority of the studies in this review focused on the mental, emotional, and other psychosocial effects of disclosure, since this is one of the reasons often cited for both disclosure and non-disclosure to children. Five of the 11 studies on mental health reported a negative impact of disclosure on some aspect of mental health (14, 17, 28, 29, 32), while two showed disclosure had a positive impact (27, 33). The remaining four studies either showed minimal, short-term negative impact or no impact (4, 26, 30, 31). Only three of the studies (two in the US and from the same PACTG 219 C prospective study and one from Kenya) had sufficiently large sample sizes (14, 26, 28), but both US studies reached differing conclusions. While the Butler et al. (26) study of 2,423 visits of 395 CLHIV did not show any statistically significant difference between pre and post HIV disclosure on QoL (general health perception; symptom distress; psychological status and physical functioning; social/role functioning and health care utilization), Gaughan et al. (28) showed in their study of 2,298 CLHIV and 1,021 children not living with HIV that knowledge of HIV status was significantly associated with increased risk of psychiatric hospitalization, with CLHIV who were aware of their status being six times more likely to be hospitalized due to psychiatric illnesses, compared to CLHIV not aware of their status (hazard ratio 6.13). It is not clear what the reasons could be for the different conclusions from the two studies. A possible explanation could be that while Butler et al. (26) measured pre- and post-disclosure QoL changes in the same CLHIV, Gaughan’s study compared psychiatric hospitalization in HIV disclosed CLHIV to children not living with HIV. The experience of a significant life event (such as death in the family and beginning school) also contributed to the positive correlation between disclosure and hospitalization and may partly explain the contrasting conclusions from the studies. Menon et al. (32) suggest that disclosure did not have a negative impact on mental health. On the contrary, their study suggests that disclosed children may have better mental outcomes than non-disclosed peers. Although the Kenya study by Vreeman et al. (14) had a large sample of 792 and reported higher rates of depression and stigma among disclosed children, and the study was not designed to assess the impact by pre- and post-disclosure characteristics. These different findings underscore the need for longitudinal and more rigorous studies.

The Uganda study on the impact of disclosure on SRH outcome showed a positive and statistically significant correlation between disclosure and condom use and contraceptive use rates that are even much higher than the general population rates for adolescents, in addition to status disclosure to partners (20). However, Battles and Wiener (29) reported that disclosed CLHIV were sexually active—with and without using condoms. The authors, incidentally, did not state how this differed from the general US population, or non-disclosed children. Obviously, this is a less well researched area and further studies are needed (20), since it is important for control of the pandemic.

Finally, a child’s knowledge of their HIV status, and the child’s subsequent disclosure of their status to others (friends and sexual partners) had an effect on child’s health outcomes. Two studies showed a positive correlation between child’s disclosure of their status on the child’s health outcome, such as increase in CD4 percent (34), increased self-competence, and decrease in problem behavior (29). However, the children’s knowledge of their serostatus did not necessarily result in high rates of status disclosure to sexual partner (20, 33): an observation that has important consequences for HIV sexual prevention efforts and HIV control.

### Implications and Recommendations

Disclosure of a child’s HIV status to the child has value in terms of positive health outcomes for the child, such as better adherence and slower disease progression (24). Yet, there does not seem to be a systematic or coherent system for child disclosure in SSA or globally. One recommendation from this review, therefore, is the need for government and program policies and guidelines that will promote child HIV disclosure in order to address the current low rates of disclosure in SSA where most CLHIV live. It is encouraging that some SSA countries have developed pediatric disclosure guidelines—either as stand-alone documents or embedded in other guideline documents (36). However, it is important that these guidelines provide enough information that will enable health care workers and/or parents/caregivers to effectively disclose to HIV-infected children. WHO has also published the *Guideline on HIV Counseling for Children up to 12 years of Age* for adaptation in countries (5). Existing pediatric disclosure models and tools, such as children’s books, videos, job aides, and curricula, aim to assist health care providers, caregivers, and/or children in disclosure (37–39). These models and tools also address some of the health outcomes identified in this review. For instance, the SANKOFA disclosure model, which is family-centered, clinic-based, and health worker facilitated, addresses adherence, viral and immunologic markers, and mental health outcomes (39).

As many CLHIV are of school age, such policies and guidelines also need to include disclosure to education personnel in the school environment, as well as how to build capacity in the school environment to limit stigma and facilitate support for CLHIV in schools. Ensuring the child’s well-being, doing no harm and reducing stigma should be important components of school-related disclosure. Although the decision to inform schools of the child’s HIV status should remain a family decision, providers and program managers can facilitate the process and help build family capacity to do this (25).

Disclosure may not always be beneficial, as negative effects may manifest both in the short and longer term, such as precipitated psychiatric issues (28). While it is not clear how much of a challenge this is in SSA, or whether the resulting psychiatric illness is due to HIV or other psychosocial factors, clinicians need to set up systems to monitor and identify warning signs of psychiatric illness and establish systems for referrals for mental health services (28). Programs that not only address clinical needs of children but also other aspects of child well-being, including psychosocial, life skills, for instance, self-competence, and SRH needs, as well as psychosocial support programs for caregivers are also needed. Programs that adequately address the SRH needs of CLHIV are a clear need from this review, especially as many CLHIV are growing into adolescence and beginning sexual activity. It is critical to reorient health care providers to address their ability and willingness to provide information and services for HIV prevention and contraceptives to CLHIV in a culturally sensitive manner. They also need to emphasize status disclosure, especially in discordant relationships (where one partner is not living with HIV), and encourage consistent condom use to prevent further infection of CLHIV and others (20).

### Research Gap

Only five of the 15 studies included in this review were conducted in SSA (none in West Africa), and two of which had very small sample size of 8 and 25 (4, 17) and limited the ability to perform rigorous analyses that would also focus on causality and not just correlations. However, currently 90% of CLHIV live in SSA (21). Clearly, therefore, a major recommendation is the need for more studies on SSA, especially as the different cultural, social, and economic environment in SSA may (or may not) influence health outcomes and HIV disclosure differently. Another recommendation is for more longitudinal studies of larger sample size, to allow more rigorous analyses, such as determining causality—not only in SSA but also in other regions (the US and elsewhere), as nine of the 15 studies reviewed were of sample size 100 or less.

Most studies in this review focused mainly on children infected perinatally. However, it is not clear if there are differences in health and related outcomes between perinatally acquired HIV and non-parental transmission (such as blood transfusion and sexual transmission) and differences in disclosure and health outcomes. Experiences of youth who learn of their status inadvertently (i.e., unintended disclosure) also need to be studied. Programs also need a better understanding of disclosure on school attendance and performance and to study the complex social needs of HIV-positive children in the school environment (25) and how programs can support CLHIV and their parents for disclosure in schools in a sensitive manner and without stigma backlash.

Current studies have limited information on the disclosure process and context. There is need for a better understanding of the appropriate process, context, and child’s age for disclosure of status and how these impact on health outcomes (4). The WHO pediatric disclosure guidance also recommends further research on who is best positioned to disclose to the child; and what factors can promote or act as barriers to disclosure (5). Such studies could provide important information for policy development and guidelines on pediatric HIV disclosure. Studies of physical health outcomes also need to include other markers of HIV disease progression, such as viral load, clinical status, and growth velocity (24). Furthermore, more studies adapted for SSA are needed that use standardized measures to assess emotional health.

### Limitations

This review and the interpretation of the findings presented here have several limitations. First, only one database was searched. It is likely that widening the study search to additional databases, such as PubMed, would have yielded other relevant studies. Also, the context of this review required only one reviewer. Thus, the study review process did not benefit from a second opinion where there were uncertainties on whether to include a study or not. The third limitation is the very small sample size of most of the studies. This limited the sophistication of analyses that could be performed by the researchers, including adjusting for confounders. As such, very limited conclusions can be drawn from the studies. Fourth, most of the studies were cross-sectional. Therefore, only correlational inferences between disclosure and health outcomes could be made, without establishing causality.

Fifth, key terms were not defined in most studies. While a few studies used standard tools developed and tested for psychometric studies (26, 28, 31, 32), in majority of the studies, it was up to the investigator to determine how anxiety, depression, and other key terms were defined and conceptualized in the studies. While CD4 count (and percent) was used as a key indicator for disease progression, inclusion of other indicators, such as the number and severity of adverse health events, viral load and growth velocity, as stronger indicators of HIV disease progression, would have made the studies better (24, 34). Finally, most of the studies included in the review were conducted outside SSA. It is not clear if similar findings would be obtained if the studies were repeated within the SSA context. These gaps notwithstanding, the findings reported in this review provide useful information for policy makers in SSA as they explore and develop pediatric disclosure guidelines. Key factors to consider in adopting these findings will include local culture and family dynamics, country resources, education, and health literacy, which can differ significantly across countries.

## Conclusion

This review highlights that HIV disclosure to CLHIV does have an effect on health and related outcomes—physical/physiological, psychological, treatment adherence, SRH, and status disclosure to others—albeit the different studies did not always reach the same conclusions, and some studies suggest disclosure may have negative outcomes. There is a very clear need for more studies on SSA, the region where the majority of CLHIV resides, as well as more rigorous and longitudinal studies, with larger study samples that will allow more sophisticated analyses that can establish causality. Information from these studies would also be valuable to countries and program managers to develop HIV disclosure policies and guidelines and programs that improve the well-being of CLHIV and their caregivers.

## Author Contributions

The author confirms being the sole contributor of this work and approved it for publication.

## Conflict of Interest Statement

The author declares that the research was conducted in the absence of any commercial or financial relationships that could be construed as a potential conflict of interest.

## Abbreviations

AIDS, acquired immune deficiency syndrome; ART, antiretroviral therapy; ARV, antiretroviral drug; CLHIV, children living with HIV; HCW, health care worker; HIV, human immunodeficiency virus; PMTCT, prevention of mother-to-child transmission of HIV; QoL, quality of life; SDQ, strengths and difficulties questionnaire; SRH, sexual and reproductive health; SSA, sub-Saharan Africa.
